# Building a robust backbone for *Astragalus* using a clade‐specific target enrichment bait set

**DOI:** 10.1002/ajb2.70084

**Published:** 2025-08-19

**Authors:** Daniele Buono, Gudrun Kadereit, Aaron Liston, Shahin Zarre, Diego F. Morales‐Briones

**Affiliations:** ^1^ Prinzessin Therese von Bayern‐Lehrstuhl für Systematik, Biodiversität und Evolution der Pflanzen, Ludwig‐Maximilians‐Universität München Munich Germany; ^2^ Staatliche Naturwissenschaftliche Sammlungen Bayerns (SNSB): Botanische Staatssammlung München, Botanischer Garten München‐Nymphenburg Munich Germany; ^3^ Department of Botany and Plant Pathology Oregon State University Corvallis OR USA; ^4^ Department of Plant Sciences, School of Biology, College of Science University of Tehran Tehran Iran

**Keywords:** *Astragalus*, cytonuclear discordance, Fabaceae, gene discordance, herbarium, mega‐genus, Papilionoideae, phylogeny, target enrichment

## Abstract

**Premise:**

With over 3100 species, *Astragalus* L. (Fabaceae) has long fascinated botanists as the largest genus of flowering plants. With an origin in the Middle Miocene, *Astragalus* has one of the highest diversification rates known in flowering plants. Comprehensive taxonomic treatments exist, and the genus is currently subdivided into 136 sections in the eastern hemisphere and 93 sections in the western hemisphere based on morphological characters. Despite considerable efforts, a comprehensive and well‐resolved phylogeny of the genus is still lacking.

**Methods:**

Here, we reconstruct the backbone phylogeny of *Astragalus* using a custom bait set capturing 819 loci specifically designed for a target enrichment approach in the Astragalean clade. We carefully selected a set of 107 taxa representing all major clades currently recognized in *Astragalus*. Of those, 80 newly sequenced taxa were obtained from herbarium specimens as old as 110 years.

**Results:**

We retrieved all the targeted loci and additional off‐target plastome sequences for all samples, including the 80 herbarium specimens. Our phylogenetic analysis reinforced the currently accepted backbone phylogeny of *Astragalus* with high support and novel details, additionally providing insights into cytonuclear phylogenetic conflicts in the genus. Evidence for potential reticulate evolution was found, providing a possible explanation for the conflicts observed.

**Conclusions:**

This work represents an important milestone in obtaining a comprehensive, herbarium‐based phylogeny of *Astragalus*, which will constitute the base to study a wealth of relevant biological questions, for example, the still unanswered question of what drove the rapid diversification of *Astragalus*, with important repercussions on our understanding of diversification in natural contexts.

*Astragalus* L. (Fabaceae, subfamily Papilionoideae), with about 3100 species (POWO, 2024), is considered the largest genus of flowering plants and represents a striking example of rapid radiation (Azani et al., 2019; Moonlight et al., 2024). The genus has a nearly worldwide distribution, occurring mainly in semiarid regions of the northern hemisphere, with about 2600 species in the eastern hemisphere (Old World) and about 500 species in the western hemisphere (New World) (Gómez‐Sosa, 1979; Welsh, 2007; Podlech and Zarre, 2013). The distribution areas of *Astragalus* species range from continent‐wide distributions—such as 13 species with a circumboreal range—to highly restricted geographic ranges for hundreds of narrow endemics (Podlech and Zarre, 2013). Of 109 *Astragalus* species assessed by the IUCN Red List, 45.9% are considered vulnerable, endangered, or critically endangered (IUCN, 2024). There are several centers of diversity of the genus, occurring in cool to warm arid or semiarid and mountainous regions in the northern hemisphere (Folk et al., 2024b). The Irano‐Turanian floristic region hosts more than 1500 species, representing the major center of diversity (Maassoumi, 1998). Iran has extensive endemism (about 620 species of 900 total are endemic), including a neoendemism and two paleoendemism areas (Maassoumi and Ashouri, 2022; Folk et al., 2024b). Other important centers of diversity are western North America with ~450 species and the Andes in South America with ~110 species (Wojciechowski et al., 1999; Wojciechowski, 2005; Scherson et al., 2008). *Astragalus* includes morphologically diverse groups of annuals (~120 spp., Azani et al., 2017) to perennial herbs (~2500 spp.) and spiny cushions (~300 spp., Podlech and Zarre, 2013). Despite its ubiquity and striking diversity, the genus is relatively poorly studied, and its phylogeny is yet to be explored comprehensively (Su et al., 2021; but see Azani et al., 2017, 2019; Folk et al., 2024a, discussed below).

## Species diversity in *Astragalus*

*Astragalus* does not have higher rates of diversification than its relatives in the Astragalean clade (e.g., genus *Oxytropis* DC. with about 600 species; POWO, 2024) or compared to other temperate papilionoid legumes. Instead, the Astragalean clade (sensu Sanderson and Liston, 1995) as a whole (ca. 3900 species) is significantly more diverse than its closest relatives (e.g., *Caragana* Lam., *Colophaca* Fisch., *Chesneya* Lindl. ex Engl., and *Guedenstaedtia* Fisch.) (Sanderson and Wojciechowski, 1996; Koenen et al., 2013). There seem to be no distinctive ecological traits in the Astragalean clade that could be regarded as or correlated with any obvious key innovation that would explain the diversification observed. Therefore, Sanderson and Wojciechowski (1996) hypothesized that demographic factors such as population fragmentation and isolation may better explain diversification within this group. Furthermore, ecological specialization in *Astragalus* to edaphic conditions and extreme microhabitats unfavorable for most other plants suggests that adaptation to local conditions may lead to divergence and persistence of lineages (Kenicer, 2005; Rundel et al., 2015; Maassoumi and Khajoei Nasab, 2023). Scherson et al. (2008) indicated that new ecological opportunities caused by the formation of different environments and microclimates as a result of the uplift of the Andes during the Pliocene (2–4 Mya) promoted the high diversification rates of *Astragalus* observed in the Southern Andes. There is an astonishing parallel to the genus *Lupinus* in which Andean radiation took place in the same period and due to similar diversification mechanisms (Hughes and Eastwood, 2006). Interestingly, the species diversity of *Astragalus* decreases toward the Northern Andes, with no records of the genus north of Ecuador in South America. Similarly, Azani et al. (2019) hypothesized that the intense uplift of the Qinghai‐Tibetan Plateau (QTP) during the Late Miocene resulted in prolonged droughts, promoting the formation of semidesert habitats, which might have favored the diversification of *Astragalus*. On the other hand, Hardion et al. (2016) supported the hypothesis that the main driver of diversification in the Mediterranean xerophytic *Astragalus* sect. *Tragacantha* DC. is range fragmentation rather than a coastal‐mountain ecological shift. The underlying causes of the high diversity might be a mix of common intrinsic traits and different environmental and geographic settings among clades and regions, which makes a macroevolutionary study of this fascinating genus highly desirable.

The origin of the genus has been dated to the Middle Miocene, 16 million years ago (Mya; 12.27–20.76 Mya), and placed in West Asia based on nrDNA (ITS) and cpDNA (*trnK/matK* + *ycf1*) data (Azani et al., 2019). A rapid diversification followed, starting from about 14 Mya, with subsequent range expansion repeatedly in the Mediterranean area and then North America (Azani et al., 2019). There exists a strong relationship between chromosome number and geographic distribution. Most eastern hemisphere and circumboreal species have euploid chromosome numbers based on *n* = 8, with *n* = 8, 16, 32, 48, 64, and 96, with common polyploids. Almost all species from the Americas (including both North and South American taxa) form the Neo‐*Astragalus* clade, which is characterized by an aneuploid chromosome number of *n* = 11, 12, 13, 14, and 15, with the exception of approximately 15 North American species (Wojciechowski et al., 1993, 1999). The origin of the aneuploid American *Astragalus* is still unresolved, with Azani et al. (2017) suggesting a Mediterranean origin of around 4.36 Mya, while the study of Su et al. (2021) supported an Asian origin instead. Azani et al. (2019) suggested a single origin of the western hemisphere *Astragalus* aneuploid species (Neo‐*Astragalus*) from within the Diholcos clade and separate origins of the euploid western hemisphere species in the Hamosa clade, and probably also in the Phaca and Hypoglottis clades (see Wojciechowski et al., 1999; Kazempour Osaloo et al., 2003, 2005).

## Current subgeneric classifications and their limitations

*Astragalus* species are classified into 136 sections in the eastern hemisphere (Podlech and Zarre, 2013) and 93 sections in the western hemisphere (Barneby, 1964) based on morphological characters. Molecular phylogenetic studies demonstrated that the most important morphological features used in classical taxonomic studies (e.g., medifixed vs. basifixed indumentum, perennial vs. annual) are homoplasious and have originated several times independently (Zarre and Azani, 2013). In *Astragalus*, 11 major clades were recovered in a phylogenetic tree by Azani et al. (2017) based on nuclear ITS + plastid *trnK*/*matK*. Su et al. (2021), based on 65 coding DNA sequences (CDS) of 117 plastomes, recovered an additional clade and referred to it as Pseudosesbanella. Otherwise, their phylogenetic tree mostly overlapped that of Azani et al. (2017), even though it lacked samples from the Ophiocarpus clade. Folk et al. (2024a) published the most recent molecular phylogenetic study, including a larger western hemisphere *Astragalus* taxon sampling. The authors included about 900 species (373 taxa belonging to the western hemisphere [~45% of the total], and 474 to the eastern hemisphere [~20% of the total]). Their phylogeny was based on a selection of approximately 100 loci (“NitFix loci”; Kates et al., 2024) intended to cover the entire nitrogen‐fixing clade. The authors recovered a phylogeny with most backbone nodes having high support except for the Astragalean clade (local posterior probability [LPP] = 0.08) and very low support and poorly resolved relationships within the main clades (average LPP = 0.605). Therefore, relationships among major clades inside *Astragalus* and the tempo of their evolution remain unclear, highlighting the need for further molecular studies based on additional data.

## Goal of the study

In this study, we used a bait set comprising 819 loci for target enrichment designed on the Astragalean clade (described by Buono et al., in press) to reconstruct a backbone phylogeny of *Astragalus* and resolve infrageneric relationships. Our DNA samples were extracted from herbarium specimens as old as 110 years, covering many of the sections currently supported by molecular data in the genus, plus several species representing other genera in the Astragalean clade. This newly produced data set was integrated with publicly available published transcriptome data. The newly produced phylogeny was compared with currently accepted and newly proposed phylogenies of *Astragalus* (Azani et al., 2017, 2019; Folk et al., 2024a). We additionally explored the possibility of reconstructing an organelle phylogeny using off‐target regions, providing novel insights into cytonuclear phylogenetic conflicts in the Astragalean clade.

## MATERIALS AND METHODS

### Taxon sampling

To compare our phylogeny with previous ones and to cover the major clades recognized in *Astragalus*, we chose to sample species from the major clades obtained in the phylogenies of Azani et al. (2017), Su et al. (2021), and Folk et al. (2024a). When specimens were available at the Botanical State Collection Munich (herbaria M and MSB), sampled vouchers were identical to those of Azani et al. (2017; Appendix S1: Table S1). To increase the representativeness of the Astragalean clade and add outgroups to our data set, we added additional taxa for which transcriptome (Zhao et al., 2021) and genomic data were available online (Table 1).

**Table 1:** Samples of *Astragalus* and other species from the Astragalean clade used in this study. In the column Sequence origin, hebarium_extraction = newly generated sequences, TRP_Zhao_et_al._2021 = transcriptome data from Zhao et al. (2021); a DOI is provided for sequences from other studies.

ID specimen	Taxon	Sequence origin	Voucher	Munich Herbarium barcode	Country	Section (Podlech & Zarre, 2013)	Year of collection	SRA accession
Ast_acaulis_1	*Astragalus acaulis* Baker	herbarium_extraction	Boufford et al., 42021	MSB‐156266	China	*Hookeriana*	2009	SAMN48979016
Ast_alopecurus_2	*A. alopecurus* Pall.	herbarium_extraction	Merxmüller & Zollitsch, 26349	M‐0336721	France	*Alopecuroidei*	1970	SAMN48979017
Ast_alyssoides_3	*A. alyssoides* Lam.	herbarium_extraction	Safavi & Alizade & Nikchehre, 84119	MSB‐110283	Iran	*Hololeuce*	2003	SAMN48979018
Ast_annularis_4	*A. annularis* Forssk.	herbarium_extraction	K. Tilbörger, s.n.	M‐0336507	Israel	*Annulares*	1992	SAMN48979019
Ast_apricus_5	*A. mirabilis* subsp. *chodshamastonicus* Rassulova	herbarium_extraction	Salmaki et al., 39875	MSB‐155300	Iran	*Caprini*	2009	SAMN48979020
Ast_arbuscula_trp	*A. arbuscula* Pall.	TRP_Zhao_et_al._2021	FC483			*Dissitiflori*		
Ast_australis_6	*A. australis* (L.) Lam.	herbarium_extraction	W. Lippert, 28900	M‐0337609	Austria	*Hemiphragmium*	2002	SAMN48979021
Ast_austroaegaeus_7	*A. austroaegaeus* Rech.f.	herbarium_extraction	N. Böhling & T. Raus 7394	M‐0155958	Greece	*Malacothrix*	1998	SAMN48979022
Ast_bhotanensis_trp	*A. bhotanensis* Baker	TRP_Zhao_et_al._2021	HM2527			*Uliginosi*		
Ast_bisulcatus_var_hay_8	*A. bisulcatus* var. *haydenianus* (A.Gray ex Brandegee) M.E.Jones	herbarium_extraction	A. Tiehm, 13563	M‐0346678	USA	Neo‐*Astragalus*	2001	SAMN48979023
Ast_bombycinus_9	*A. bombycinus* Boiss.	herbarium_extraction	Rechinger, 9857	M‐0292722	Iraq	*Platyglottis*	1957	SAMN48979024
Ast_brachypetalus_10	*A. brachypetalus* Trautv.	herbarium_extraction	H. Akhani, 9390	M‐0292725	Iran	*Hypoglottidei*	1994	SAMN48979025
Ast_canadensis_var_bre_11	*A. canadensis* var. *brevidens* (Gand.) Barneby	herbarium_extraction	A. Tiehm, 13225	MSB‐186811	USA	*Uliginosi*	2000	SAMN48979026
Ast_caprinus_12	*A. caprinus* subsp. *caprinus* L.	herbarium_extraction	O. Anders, 2774	MSB‐186824	Iraq	*Caprini*	1969	SAMN48979027
Ast_caraganae_13	*A. caraganae* Fisch. & C.A.Mey.	herbarium_extraction	American‐Iranian Botanical Delegation 33854 TUH			*Astragalus*		SAMN48979028
Ast_chorizanthus_15	*A. chorizanthus* Rech.f. & Gilli	herbarium_extraction	D. Podlech, 11026	M‐0123827	Afghanistan	*Dissitiflori*	1965	SAMN48979029
Ast_christianus_sub_chr_16	*A. christianus* subsp. *christianus* L.	herbarium_extraction	M. Nydegger, 47424	MSB‐186827	Turkey	*Astragalus*	1993	SAMN48979030
Ast_chrysochlorus_trp	*A. chrysochlorus* Boiss. & Kotschy	TRP_Zhao_et_al._2021	SRR2107187			*Onobrychoidei*		
Ast_contortuplicatus_17	*A. contortuplicatus* L.	herbarium_extraction	Botschkin & Klinkova, 15188	MSB‐186832	Russia‐Europe	*Cycloglottis*	1989	SAMN48979031
Ast_cornu_18	*A. cornu‐bovis* Lipsky	herbarium_extraction	K. H. Rechinger, 51405	M‐0257255	Iran	*Sesamei*	1975	SAMN48979032
Ast_dactylocarpus_19	*A. dactylocarpus* Boiss.	herbarium_extraction	H. Akhani, 5572	MSB‐188989	Iran	*Chronopus*	1989	SAMN48979033
Ast_depressus_20	*A. depressus* L.	herbarium_extraction	W. Lang, s.n.	M‐0292727	Switzerland	*Tapinodes*	1988	SAMN48979034
Ast_dipelta_21	*A. dipelta* Bunge	herbarium_extraction	Assadi & Maassoumi, 50863	MSB‐186834	Iran	*Dipelta*	1984	SAMN48979035
Ast_dolichopyllus_22	*A. dolichophyllus* Pall.	herbarium_extraction	A. Sytin	MSB‐189195	Ukraine	*Trachycercis*	2000	SAMN48979036
Ast_echinatus_23	*A. echinatus* Murr.	herbarium_extraction	D. Podlech, 51232	MSB‐002549	Spain	*Pentaglottis*	1996	SAMN48979037
Ast_echinops_24	*A. echinops* Aucher ex Boiss.	herbarium_extraction	D. Podlech et al. 55236	MSB‐194125	Iran	*Alopecuroidei*	2001	SAMN48979038
Ast_epiglottis_25	*A. epiglottis* L.	herbarium_extraction	D. Podlech, 45878	MSB‐186837	Morocco	*Epiglottis*	1989	SAMN48979039
Ast_eremophilus_sub_ere_26	*A. eremophilus* subsp. *eremophilus* Boiss.	herbarium_extraction	Maassoumi & Abouhamzeh, 52074	MSB‐005978	Iran	*Harpilobus*	1985	SAMN48979040
Ast_falconeri_27	*A. falconeri* Bunge	herbarium_extraction	E. Eberhadt 7650	MSB‐142396	Pakistan	*Pseudosesbanella*	2000	SAMN48979041
Ast_filicaulis_28	*A. filicaulis* Fisch. & C.A.Mey. ex Ledeb.	herbarium_extraction	Assadi & Maassoumi, 55373	MSB‐186839	Iran	*Sesamei*	1986	SAMN48979042
Ast_frigidus_29	*A. frigidus* (L.) A.Gray	herbarium_extraction	D. Podlech 9964	MSB‐194361	Austria	*Cenantrum*	1964	SAMN48979043
Ast_fruticosus_30	*A. fruticosus* Forssk.	herbarium_extraction	D. Podlech et al. 29825	MSB‐001979	Afghanistan	*Chronopus*	1977	SAMN48979044
Ast_glycyphylloides_31	*A. glycyphylloides* DC.	herbarium_extraction	A. Ghahreman & V. Mozaffarian, 9661	MSB‐186843	Iran	*Glycyphyllus*	1990	SAMN48979045
Ast_guttatus_32	*A. guttatus* Banks & Sol.	herbarium_extraction	Sytin	MSB‐002777	Ukraine	*Aulacolobus*	2000	SAMN48979046
Ast_hemiphaca_33	*A. hemiphaca* Kar. & Kir.	herbarium_extraction	V. Sapozhnikov & B. Shishkin.	MSB‐186849	Kazakhstan	*Oroboidei*	1912	SAMN48979047
Ast_himalayanus_14	A. himalayanus Klotzsch	herbarium_extraction	U. Schickhoff 1180	MSB‐152970	Pakistan	*Chlorostachys*	1990	SAMN48979048
Ast_hispidulus_34	*A. hispidulus* DC.	herbarium_extraction	D. Podlech, 44568	MSB‐005977	Egypt	*Hispiduli*	1989	SAMN48979049
Ast_hissaricus_35	*A. hissaricus* Lipsky	herbarium_extraction	Varivtseva & Nepli, 517	MSB‐186847	Tajikistan	*Hololeuce*	1948	SAMN48979050
Ast_hoffmeisteri_36	*A. hoffmeisteri* (Klotzsch) Ali	herbarium_extraction	Eberhadt 9357	MSB‐142177	Pakistan	*Pseudosesbanella*	2000	SAMN48979051
Ast_hymenostegis_37	*A. hymenostegis* Fisch. & C.A.Mey.	herbarium_extraction	Bagheri, 98071	MSB‐168221	Azerbaijan	*Hymenostegis*	2013	SAMN48979052
Ast_incanus_38	*A. incanus* subsp. *incanus* L.	herbarium_extraction	Lambinon	MSB‐139789	Spain	*Incani*	1985	SAMN48979053
Ast_intercedens_39	*A. intercedens* Sam. ex Rech.f.	herbarium_extraction	Colonette, 6513	MSB‐186851	Saudi Arabia	*Hispiduli*	1988	SAMN48979054
Ast_laxmannii_trp	*A. laxmannii* Jacq.	TRP_Zhao_et_al._2021	HM2518			*Onobrychoidei*		
Ast_lentiginosus_trp	*A. lentiginosus* Douglas ex Hook.	TRP_Zhao_et_al._2021	HM3017			Neo‐*Astragalus*		
Ast_leucocephalus_40	*A. leucocephalus* Graham	herbarium_extraction	Schickhoff, 260	MSB‐142142	Pakistan	*Poliothrix*	1990	SAMN48979055
Ast_lipskyi_41	*A. lipskyi* Popov	herbarium_extraction	D. Podlech 413	MSB‐191251	Uzbekistan	*Caprini*	1967	SAMN48979056
Ast_lonchocarpus_42	*A. lonchocarpus* Torr.	herbarium_extraction	N.H. Holmegren, 11924	M‐0346679	USA	Neo‐*Astragalus*	1993	SAMN48979057
Ast_macrobotrys_43	*A. macrobotrys* Bunge	herbarium_extraction	Ghahreman et al. 28178	MSB‐002066	Iran	*Ammodendron*	2002	SAMN48979058
Ast_macrotropis_44	*A. macrotropis* Bunge	herbarium_extraction	Mursaliev et al.	MSB‐191448	Kirgizstan	*Dissitiflori*	1967	SAMN48979059
Ast_macrourus_45	*A. macrourus* Fisch. & C.A.Mey.	herbarium_extraction	Podlech & Maassoumi & Zarre, 55251	MSB‐001838	Iran	*Malacothrix*	2001	SAMN48979060
Ast_mareoticus_46	*A. mareoticus* Delile	herbarium_extraction	D. Podlech, 49256	MSB‐186855	Morocco	*Harpilobus*	1990	SAMN48979061
Ast_membranaceus_trp	*A. membranaceus* Fisch. ex Bunge	TRP_Zhao_et_al._2021	HJMP_1KP			*Cenantrum*		
Ast_microcephalus_47	*A. microcephalus* Willd.	herbarium_extraction	D. Podlech, 55200	MSB‐186857	Iran	*Rhacophorus*	2001	SAMN48979062
Ast_mirabilis_sub_cho_48	*A. mirabilis* subsp. *chodshamastonicus* Rassulova	herbarium_extraction	M. R. Rassulova et al.	M‐0337892	Tadjikistan	*Pendulina*	1976	SAMN48979063
Ast_monspessulanus_49	*A. monspessulanus* L.	herbarium_extraction	Hertel 38.972	M‐0346681	Italy	*Incani*	1997	SAMN48979064
Ast_mucronifolius_50	*A. mucronifolius* Boiss.	herbarium_extraction	Ghahreman et al. 21576	MSB‐001782	Iran	*Leucocercis*	1998	SAMN48979065
Ast_norvegicus_51	*A. norvegicus* Weber	herbarium_extraction	Buttler and Gauhl, 8260	MSB‐186858	Norway	*Oroboidei*	1965	SAMN48979066
Ast_nothoxys_52	*A. nothoxys* A.Gray	herbarium_extraction	A. Meebold, 15315	M‐0336502	USA	Neo‐*Astragalus*	1932	SAMN48979067
Ast_nuttallianus_53	*A. nuttallianus* DC.	herbarium_extraction	A. Meebold, 8820	M‐0346680	USA ‐ Arizona	Neo‐*Astragalus*	1930	SAMN48979068
Ast_olgae_54	*A. olgae* Bunge	herbarium_extraction	W. Lipsky	MSB‐194428	Tadzhikistan	*Pelta*	1899	SAMN48979069
Ast_ophiocarpus_55	*A. ophiocarpus* Bunge	herbarium_extraction	D. Podlech, 30832	MSB‐186859	Iran	*Ophiocarpus*	1978	SAMN48979070
Ast_oreades_56	*A. oreades* C.A.Mey.	herbarium_extraction	Ekici, 3768	MSB‐146896	Turkey	*Hypoglottidei*	2007	SAMN48979071
Ast_oxyodon_57	*A. oxyodon* Baker	herbarium_extraction	A. Millinger, 3509	MSB‐186862	Pakistan	*Komaroviella*	1999	SAMN48979072
Ast_pelecinus_sub_pel_58	*A. pelecinus* subsp. *pelecinus* L.	herbarium_extraction	D. Podlech, 51802	MSB‐005973	Spain	*Biserrula*	1993	SAMN48979073
Ast_penetratus_59	*A. penetratus* Maassoumi	herbarium_extraction	Parishani, 14197	M‐0336510	Iran	*Brachylobium*	2003	SAMN48979074
Ast_polycladus_61	*A. polycladus* Bureau & Franch.	herbarium_extraction	Dickoré, 14096	MSB‐186863	China	*Poliothrix*	1996	SAMN48979075
Ast_propinquus_trp	*A. propinquus* Schischk.	TRP_Zhao_et_al._2021	MYMP_1KP			*Trachycercis*		
Ast_psilacanthus_62	*A. psilacanthus* Benth.	herbarium_extraction	Don Bedunah 29	MSB‐150724	Afghanistan	*Aegacantha*	2008	SAMN48979076
Ast_raphaelis_63	*A. raphaelis* G.Ferro	herbarium_extraction	Brullo & Giusso & Sciandrello	MSB‐138169	Italy	*Sesamei*	2006	SAMN48979077
Ast_scaberrimus_trp	*A. scaberrimus* Bunge	TRP_Zhao_et_al._2021	HM2508			*Trachycercis*		
Ast_scheremetevianus_64	*A. scheremetevianus* O.Fedtsch.	herbarium_extraction	D. E. Boufford, 41175, MSB	MSB‐139591	Tajikistan	*Scheremeteviana*	2002	SAMN48979078
Ast_schimperi_65	*A. schimperi* Boiss.	herbarium_extraction	A. Danin, 10033	MSB‐186865	Israel	*Sesamei*	1988	SAMN48979079
Ast_sclerocladus_66	*A. sclerocladus* Bunge	herbarium_extraction	American‐Iranian Botanical Delegation, 33731	MSB‐116432	Iran	*Acanthophace*	2004	SAMN48979080
Ast_scorpioides_67	*A. scorpioides* Pourr. ex Willd.	herbarium_extraction	Vogt & Oberprieler, 18‐427	MSB‐005965	Morocco	*Sesamei*	1995	SAMN48979081
Ast_siculus_68	*A. siculus* Biv.	herbarium_extraction	Merxmüller & Grau 20471	M‐0334754	Italy	*Rhacophorus*	1965	SAMN48979082
Ast_simonii_69	*A. simonii* Hub.‐Mor.	herbarium_extraction	Nydegger, 46239	MSB‐186868	Turkey	*Theiochrus*	1992	SAMN48979083
Ast_stalinskyi_70	*A. stalinskyi* Širj.	herbarium_extraction	Assadi & Maassoumi, 50866	MSB‐186873	Iran	*Ankylotus*	1984	SAMN48979084
Ast_substipitatus_71	*A. substipitatus* Gontsch	herbarium_extraction	Vasak	M‐0334755	China	*Craccina*	1974	SAMN48979085
Ast_sulcatus_72	*A. sulcatus* L.	herbarium_extraction	O. Angerer	M‐0346682	Austria	*Craccina*	1984	SAMN48979086
Ast_sungpanensis_73	*A. sungpanensis* E.Peter	herbarium_extraction	D. E. Boufford, 41175, MSB	MSB‐156242	China	*Poliothrix*	2009	SAMN48979087
Ast_tatjanae_74	*A. tatjanae* Lincz.	herbarium_extraction	Rechinger & Podlech 34044	M‐0334756	Afghanistan	*Eremophysa*	1967	SAMN48979088
Ast_tortuosus_75	*A. tortuosus* DC.	herbarium_extraction	Zaree et. al. 45257	M‐0346683	Iran	*Anthylloidei*	2014	SAMN48979089
Ast_tribuloides_60	*A. tribuloides* Delile	herbarium_extraction	Zarre & Isalkmaki & Ebrahimi, 34339	MSB‐155256	Iran	*Sesamei*	2009	SAMN48979090
Ast_uliginosus_76	*A. uliginosus* L.	herbarium_extraction	Korotkova & Kovaleva, 993	MSB‐186878	Russia‐Siberia	*Uliginosi*	1977	SAMN48979091
Ast_webbianus_77	*A. webbianus* Benth.	herbarium_extraction	Huss 410b	MSB‐194429	Afghanistan	*Caprini*	1975	SAMN48979092
Alh_sparsifolia_trp	*Alhagi sparsifolia* Shap.	TRP_Zhao_et_al._2021	FC492					
Cara_sinica_trp	*Caragana sinica* (Buc'hoz) Rehder	TRP_Zhao_et_al._2021	UN0017					
Carm_subulata_trp	*Carmichaelia subulata* Kirk	TRP_Zhao_et_al._2021	FC551					
Che_polystichoides_trp	*Chesneya polystichoides* (Hand.‐Mazz.) Ali	TRP_Zhao_et_al._2021	FC530					
Cic_arietinum_gen	*Cicer arietinum* L.	https://doi.org/10.1038/nbt.2491						
Cli_puniceus_trp	*Clianthus puniceus* (G.Don) Sol. ex Lindl.	TRP_Zhao_et_al._2021	HM2728					
Col_arborescens_trp	*Colutea arborescens* L.	TRP_Zhao_et_al._2021	ZY007					
Col_persica_78	*Colutea persica* Boiss.	herbarium_extraction	D. Podlech, 970	MSB‐194427	Iran		1977	SAMN48979093
Cor_multijugum_trp	*Corethrodendron multijugum* (Maxim.) B.H.Choi & H.Ohashi	TRP_Zhao_et_al._2021	HM2563					
Ere_songoricum_trp	*Eremosparton songoricum* (Litv.) Vassilcz.	TRP_Zhao_et_al._2021	Ere					
Gue_stenophylla_trp	*Gueldenstaedtia stenophylla* Bunge	TRP_Zhao_et_al._2021	HM2307					
Hed_scoparium_trp	*Hedysarum scoparium* Fisch. & C.A.Mey.	TRP_Zhao_et_al._2021	HM2516a					
Med_truncatula_gen	*Medicago truncatula* Gaertn.	https://doi.org/10.1186/1471-2164-15-312						
Oxy_ochrocephala_trp	*Oxytropis ochrocephala* Bunge	TRP_Zhao_et_al._2021	HM2524					
Oxy_purpurea_79	*Oxytropis purpurea* Markgr.	herbarium_extraction	A.O. Chater, 303		Yugoslavia		1971	SAMN48979094
Phy_camptodontum_trp	*Phyllolobium camptodontum* (Franch.) M.L.Zhang & Podlech	TRP_Zhao_et_al._2021	HM3220					
Pod_vogelii_sub_vog_80	Podlechiella vogelii (Webb) Maassoumi & Kaz.Osaloo	herbarium_extraction	D. Podlech, 36700	M‐0342370	Algeria		1982	SAMN48979095
Sph_salsula_trp	*Sphaerophysa salsula* (Pall.) DC.	TRP_Zhao_et_al._2021	HM2561_2					
Sut_montana_trp	*Sutherlandia montana* (E.Phillips & R.A.Dyer) Goldblatt & J.C.Manning	TRP_Zhao_et_al._2021	ZY438					
Swa_canescens_trp	*Swainsona canescens* (Benth.) F.Muell.	TRP_Zhao_et_al._2021	FC278					
Tib_yunnanensis_trp	*Tibetia yunnanensis* (Franch.) H.P.Tsui	TRP_Zhao_et_al._2021	HM3240					
Tri_pratense_gen	*Trifolium pratense* L.	https://doi.org/10.1038/srep17394						

### Library preparation, target enrichment, and sequencing

DNA was extracted from ~20 mg of dry herbarium plant material using the NucleoSpin Plant II kit (Macherey‐Nagel, Düren, Germany) and the manufacturer's manual with slight modifications. Cell lysis in step 2a was performed using 600 μL buffer PL1, no RNase A was added since the plant materials were too old to preserve RNA, and incubation lasted for 1.5 h. An extra wash was used in step 6, and 350 μL buffer PW2 was added before drying the membrane completely. Finally, DNA was eluted in 50 μL buffer PE (5 mM Tris/HCl, pH 8.5). DNA concentration was measured using an Invitrogen Qubit 4 Fluorometer using the High Sensitivity (HS) assay kit (Thermo Fisher Scientific, Waltham, MA, USA), and fragmentation was visually evaluated in a 1% agarose gel.

Before library preparation, genomic DNA was diluted to 250 ng in 55 μL buffer PE and sonicated using a Covaris M220 Focused‐ultrasonicator (Covaris, Woburn, MA, USA) to obtain DNA fragments of about 350 bp. Accurate profiling of the sonicated sample size distribution was performed on an Agilent 4150 TapeStation System using the High Sensitivity D1000 ScreenTape (Agilent Technologies, Santa Clara, CA, USA). Libraries were prepared using NEBNext Ultra II DNA Library Prep Kit for Illumina and the NEBNext Multiplex Oligos for Illumina (Dual Index Primers Set 1, New England Biolabs, Ipswich, MA, USA) and following the manufacturer's protocol. In step 3 of the protocol, the size selection of adaptor‐ligated DNA was adjusted for each sample according to the average sample size measured with TapeStation. PCR amplification of the adaptor‐ligated DNA was performed with eight cycles. Each library was profiled with the Qubit and TapeStation assay, as above.

Individual libraries were mixed in 16‐sample pools, using equal DNA amounts (200 ng or 250 ng) from samples with similar fragment average sizes. Pooled libraries were dried by vacuum centrifugation and resuspended in 7 μL nuclease‐free water. Target enrichment was performed using the myBaits Hybridization Capture Kits (Daicel Arbor Biosciences, Ann Arbor, MI, USA) and the manufacturer's protocol v.5.02. A hybridization temperature T_H_ of 60°C was chosen. The wash temperature T_W_ also corresponded to 60°C. The enriched libraries were amplified with 10 PCR cycles. After amplification, pooled libraries were purified using NucleoSpin Gel and PCR Clean‐up Kit (Macherey‐Nagel, Düren, Germany) and characterized by measuring concentration and fragment sizes as described above.

The 16‐sample pools were again pooled in equimolar quantities and sequenced at the Core Facility Genomics (CF‐GEN) of the Helmholtz Zentrum München, Germany (Deutsches Forschungszentrum für Gesundheit und Umwelt, GmbH) on an Illumina Nextseq. 1000 Sequencing System.

### Nuclear data analysis

Raw data were first inspected using FastQC v.0.11.8 (Andrews, 2010) and MultiQC v.1.19 (Ewels et al., 2016). PCR duplicates (identical or nearly identical sequences with some mismatches) were removed using ParDRe v.2.2.5, leaving the number of allowed mismatches as the default setting of zero (González‐Domínguez and Schmidt, 2016). We used Trimmomatic v.0.39 (Bolger et al., 2014) to cut Illumina adapters and sequences below a quality threshold of 20 and to drop the read if less than 25 bp. Cleaned sequences were inspected again using FastQC and MultiQC to confirm the quality standards. HybPiper v.2.1.5 (Johnson et al., 2016) was used for locus assembly. The best value for SPAdes (Prjibelski et al., 2020) coverage cutoff (‐‐cov_cutoff option) was assigned individually to each sample by running a preliminary assembly with default parameters and calculating the average coverage and standard error per sample using a custom R script. The percentage identity threshold for retaining Exonerate hits (‐‐thresh option) and the percentage similarity threshold for the sliding window (‐‐exonerate_hit_sliding_window_thresh option) were both assigned to 85. The option ‐‐chimeric_stitched_contig_edit_distance was set to 0, and ‐‐chimeric_stitched_contig_discordant_reads_cutoff was set to 1. Two different target files (option ‐t_dna) were used to assemble the ingroups (which include only *Astragalus* sequences) and outgroups (which included the other Astragalean species and three outgroup species—*Medicago truncatula* Gaertn., *Trifolium pratense* L., and *Cicer arietinum* L.). Those target sequences consisted of ortholog exons obtained using the method described by Morales‐Briones et al. (2022), based on additional transcriptome data from Zhao et al. (2021) and other sequences available on NCBI. We selected the Diamond method (‐‐diamond option) to map reads to the target loci. The function paralog_retriever included in HybPiper was used to recover coding sequences from putative alternative long paralogs. Orthology inference followed the pipeline of Morales‐Briones et al. (2022). All scripts used are available at https://bitbucket.org/dfmoralesb/target_enrichment_orthology/src/master/. Sequences were aligned using MACSE v.2.07 (Ranwez et al., 2018). We used Pxclsq v.1.3 (Brown et al., 2017) with a minimum column occupancy of 0.1 (10%) to remove sites with missing data. To infer gene trees, we used IQtree v.2.3.0 (Minh et al., 2020) using standard model selection for ModelFinder (option ‐m TEST; Kalyaanamoorthy et al., 2017) and 1000 Ultrafast bootstrap replicates for node support. Tips in the gene trees that were mono‐ and paraphyletic were masked, and the tip with the most unambiguous characters in the trimmed alignment was kept. TreeShrink v.1.3.9 (Mai and Mirarab, 2018) was used to remove abnormally long branches, using a quantile value of 0.1 and excluding outgroups. Homolog fasta files were then generated from those trees and aligned using OMM MACSE v.12.01. Homolog gene trees were then generated from those sequences using IQtree. To infer orthologs, we used the monophyletic outgroup (MO) method described by Yang and Smith (2014). After aligning and cleaning the ortholog sequences as described above, the final ortholog gene trees were reconstructed using IQtree, with settings as described above. ASTRAL v1.19.4.5 was used to produce a quartet‐based species tree (Zhang et al., 2020). We also used ASTRAL‐Pro3 v1.19.3.5 to infer a species tree using homolog trees because this tool allows multicopy genes. An aligned supermatrix of fasta sequences was obtained, concatenating all the ortholog gene sequences. This supermatrix was then used to build the concatenated ML species tree using IQtree, with the same settings as specified above. To investigate gene tree discordance, we used PhyParts v.0.0.1 (Smith et al., 2015) and QuartetSampling (QS) v.1.3.1 (Pease et al., 2018). Before running PhyParts, ortholog trees were rooted using *Trifolium pratense*, *Medicago truncatula*, and *Cicer arietinum* as outgroups, and the analysis was executed two times: once with option ‐a = 1 and ‐s = 70 (to get the total of concordant and discordant nodes that passed the support threshold of 70%), and once with option ‐a = 0 (to obtain the number of nodes that did not pass the threshold of 70%). Those two results were combined using an R script and plotted in Python v.3.10.12 (Python Software Foundation, Beaverton, OR, USA) to obtain the proportion of uninformative and missing data. QuartetSampling was executed using the aligned supermatrix as input, with the option ‐g to include partitions (per locus). Results were plotted with an R script. To investigate potential reticulation relationships in the backbone of *Astragalus*, phylogenetic networks were reconstructed using PhyloNet v.3.8.2 (Wen et al., 2018). For this analysis, 22 taxa representing the 11 major clades retrieved were selected based on the highest number of ortholog sequences, resulting in a data set that included 576 loci. Sequences of those selected taxa were aligned and cleaned, and gene trees were built in the same way as described above. Species networks were then inferred using maximum pseudo‐likelihood (Yu and Nakhleh, 2015), with the number of reticulations ranging from 0 to 6. The resulting networks and inheritance probabilities were plotted using the Julia package PyPlot v2.11.6 (https://github.com/JuliaPy/PyPlot.jl; S. G. Johnson, MIT).

### Chloroplast DNA sequence assembly

Off‐target chloroplast sequences were assembled using FastPlast v.1.2.9 (McKain and Wilson, 2017). Filtered SPAdes contigs were imported into Geneious v.2023.2.1 (https://www.geneious.com) and mapped to the complete plastome of *A. pattersonii* (NC_063490, ~123kbp), selecting the option for mapping at high sensitivity. Discordant overlapping contig sequences were manually removed by keeping only the sequence that was more similar to the reference. A consensus fasta file was then extracted for each sample. Additional complete plastome sequences were downloaded from NCBI (Appendix S1: Table S2). A single fasta file, including all the sample sequences, was then produced and aligned using MAFFT v.7.453 (Katoh and Standley, 2013). The alignment was cleaned using Pxclsq, removing columns with more than 40% missing data. A maximum likelihood (ML) tree was inferred using IQtree and standard model selection. Abnormally long branching samples were removed from the final tree after visual inspection. Discordance analysis was performed using QuartetSampling. To select the most appropriate model of evolution separately for each plastome region, coding sequences (CDS) were extracted by producing a consensus alignment with the annotated *A. pattersonii* complete plastome sequence. Maximum likelihood trees were then reconstructed in IQtree with a partition table for the CDS. Another tree was reconstructed using a partition table for codon position.

## RESULTS

### Sequencing and assembling

Target enrichment data for samples representing 80 species were newly generated in this study, of which 77 belong to *Astragalus* and three to other genera in the Astragalean clade (*Colutea*, *Oxytropis*, and *Podlechiella*). The number of raw paired‐end reads ranged between 1.2 million and 12 million. The average number of loci with at least 75% length recovery was 701.1, ranging from 484 to 767 (Appendix S1: Table S3). There seemed to be no correlation between the herbarium specimen year of collection (from 1899 to 2014) and the number of loci recovered by HybPiper (Appendix S2: Figure S1). HybPiper gave an average of 33.4 paralog warnings per sample, ranging from 3 to 252 (Appendix S1: Table S3). On average, 599.8 ortholog sequences per specimen were retrieved, ranging from 230 to 718 (Appendix S1: Table S4). The final nuclear data set included 781 MO orthologs with a minimum of 20 taxa per locus. The concatenated matrix resulted in 778,623 aligned characters, with an overall matrix occupancy of 73%.

### Phylogenetic inference

**Figure 1:**
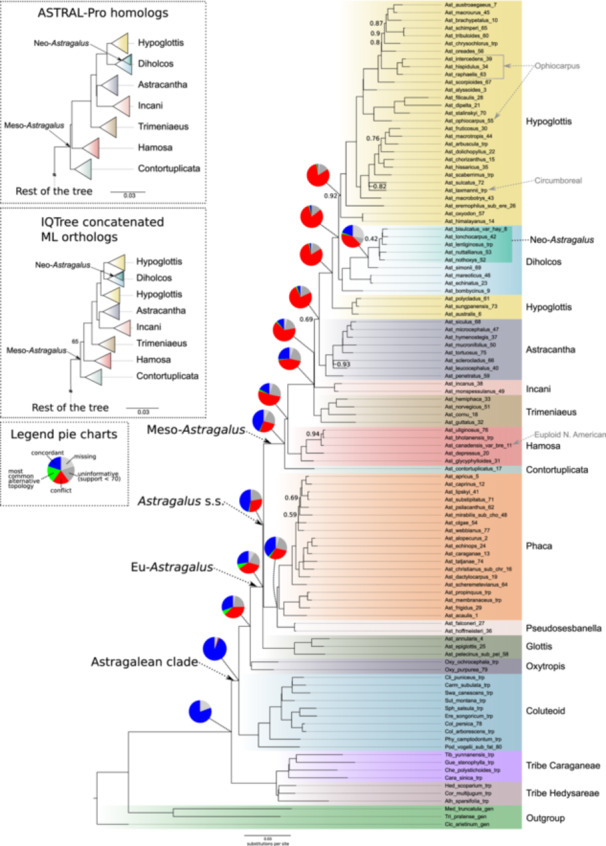
Coalescent‐based species tree of *Astragalus* obtained with ASTRAL and based on 781 orthologous loci. Node support (local posterior probability, LPP) ≥0.95 when not indicated. Pie charts indicate gene discordance at nodes along the backbone as calculated by PhyParts. Color‐coded clade names inside Eu‐*Astragalus* follow those of Azani et al. (2017) and Su et al. (2021). Inserts show major differences along the backbone using different methods as indicated, omitting parts of the trees for which the topology was identical.

**Figure 2:**
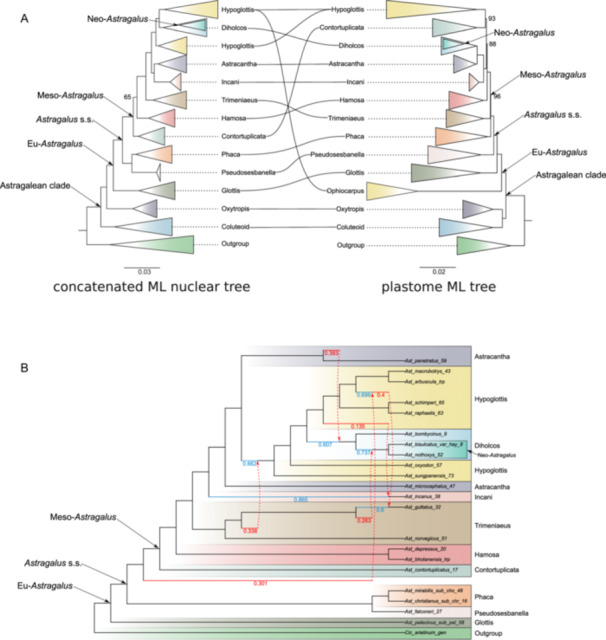
Cytonuclear discordance and nuclear phylogenetic networks suggest the presence of hybridization events in the backbone of *Astragalus*. (A) Comparison of concatenated maximum likelihood (ML) nuclear tree (left) and ML chloroplast tree (right). Ultrafast bootstrap support = 100 when not indicated. The sizes of collapsed clades in both phylogenies are not proportional to the number of taxa sampled. See Appendix S2 (Figures S2 and S3) for the extended phylogenies represented here. (B) Best phylogenetic network obtained with PhyloNet using 22 taxa representing the major clades recovered in the nuclear phylogeny. Red arrows indicate the direction of the reticulate events from the minor edge to the major edge (blue)—Numbers on the branches for reticulation events indicate inheritance probabilities.

The coalescent‐based species trees obtained with ASTRAL and ASTRAL‐Pro (Figure 1 and insert, respectively) and the concatenated ML tree obtained with IQTree (insert Figure 1; Appendix S2: Figure S2) showed very similar topologies and support values (local posterior probability [LPP] and bootstrap [BS], respectively) along the backbone, with some minor differences in the relationships of the deepest nodes (e.g., ASTRAL‐Pro produced a monophyletic Hypoglottis clade). The Astragalean clade and Eu‐*Astragalus* were recovered as monophyletic with high support (LPP = 1, BS = 100), with species placed in clades mostly consistent with previous studies (e.g., Azani et al., 2017; Su et al., 2021; Folk et al., 2024a). In Eu‐*Astragalus*, the clades Glottis, Pseudosesbanella, Phaca, Contortuplicata, Hamosa, Trimeniaeus, Incani, Astracantha, Hypoglottis, Diholcos, and Neo‐*Astragalus* were recovered with high support except for Astracantha in the coalescent tree (LPP = 0.69, BS = 100) and Hamosa in the concatenated ML tree (LPP = 1, BS = 65). However, unlike Azani et al. (2017), Diholcos was nested in Hypoglottis, splitting Hypoglottis into two fully supported clades (LPP = 1, BS = 100). Furthermore, in both our nuclear trees, the Ophiocarpus clade was dispersed and polyphyletic among Hypoglottis taxa (Figure 1; Appendix S2: Figure S2), in contrast with our chloroplast data set, which instead supported a monophyletic Ophiocarpus (BS = 100) sister to Glottis (Figure 2A; Appendix S2: Figure S3). Discordance analysis indicated strong monophyly for the Astragalean clade (628 informative genes concordant out of 628, QS score 1/–/0.96) and the Eu‐*Astragalus* + *Oxytropis* (728 informative genes concordant out of 741, QS score 1/–/0.96; Figure 1; Appendix S2: Figures S4, S5). However, along the backbone of *Astragalus* and especially for clades in Meso‐*Astragalus*, high levels of gene tree discordance and high QS skewed frequencies of alternative placement were shown (e.g., 23 informative out of 668 genes with a QS score of 0.27/1/0.48 for the node between Hypoglottis and Diholcos), despite having high support (LPP = 1, BS = 100, Figure 1; Appendix S2: Figure S2).

Phylogenetic networks generated with PhyloNet indicate the presence of extensive reticulate evolution between most of the major *Astragalus* clades (Figure 2B; Appendix S2: Figure S6). The most likely number of reticulate events was six, based on a log‐likelihood of –392,572 (Appendix S2: Figure S7). The only reticulation event recorded outside Meso‐*Astragalus* involves a ghost lineage (either not sampled or extinct) sister to the Contortuplicata and Hypoglottis clades, with an inheritance probability of 0.3 (Figure 2B). Inside Meso‐*Astragalus*, several reticulate events with inheritance probability ranging from 0.135 to 0.4 were detected between Trimeniaeus and a common ancestor of Hypoglottis plus Diholcos, Trimeniaeus and Neo‐*Astragalus*, Hypoglottis and Incani, Hypoglottis and Trimeniaeus, and Astracantha and Diholcos (Figure 2B; Appendix S2: Figure S6).

### Chloroplast DNA sequences

Even though chloroplast sequences were not directly targeted, they could be obtained for all samples. The concatenated matrix resulted in 114,580 aligned nucleotide characters, with 74.6% overall matrix occupancy. There was no appreciable difference between the trees based on the whole‐plastome data, CDS, or using different models of sequence evolution for codon position. Therefore, subsequent analyses were based on the whole plastome. The resulting ML tree showed high support along the backbone (Figure 2A right; Appendix S2: Figure S3), with a topology in agreement with a previous plastome phylogeny (Su et al., 2021). All the clades resolved in our plastome tree, except clade Ophiocarpus, agreed with our nuclear trees (both concatenated ML and coalescent‐based). Nonetheless, some were placed in different positions along the backbone (Figure 2A). Specifically, the Ophiocarpus clade was monophyletic and sister to the Glottis clade in the plastome tree, while it was split in two and nested inside the Hypoglottis clade in the nuclear trees (Figures 1, 2). Furthermore, Trimeniaeus, Contortuplicata, and Diholcos clades had different positions inside Eu‐*Astragalus*. The Hypoglottis clade was recovered as monophyletic, with the Contortuplicata clade sister to it in the plastome tree. The latter was sister to Hamosa in the nuclear trees (Figures 1, 2).

## DISCUSSION

### Reconstruction of *Astragalus* backbone phylogeny

In this study, we produced a robust backbone phylogeny of *Astragalus* by using target enrichment with a clade‐specific bait set. Our sampling, with 85 *Astragalus* species, covered about 3% of the eastern hemisphere and 1% of the western hemisphere species. Nevertheless, we selected the representatives carefully to cover all major clades currently supported by molecular data (Azani et al., 2017; Su et al., 2021; Folk et al., 2024a). We obtained an overall highly supported species tree, even though we observed large gene tree discordance and cytonuclear discordance (Figures 1, 2). Our data had low levels of missing or uninformative data (gray and dark gray pie charts in Figure 1; Appendix S2: Figure S4), excluding the low informativeness of loci selected as the main cause of this conflict. Incomplete lineage sorting, often observed in evolutionarily young clades, and reticulate evolution may instead explain the observed discordances better (Smith et al., 2015; Vatanparast et al., 2018; Morales‐Briones et al., 2021). Our phylogenetic network analysis supported several events of reticulate evolution between most of the recovered *Astragalus* clades (Figure 2B; Appendix S2: Figure S6). Thanks to the large number of loci targeted by our bait set designed specifically for the Astragalean clade, the highly supported backbone of the phylogeny fits, but partially challenges the current understanding of the genus, highlighting previously unexplored cytonuclear discrepancies. Our phylogenies recovered clades and relationships at unprecedented resolution.

### Overlap with current phylogenies

**Figure 3:**
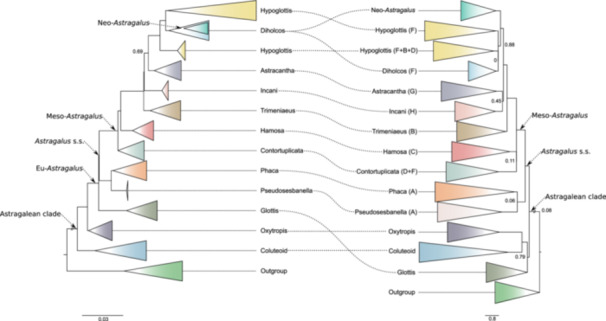
Comparison between the two coalescent‐based species trees (ASTRAL) produced in this study (left) and by Folk et al. (2024a) (right). Internal node support (local posterior probability) ≥0.95 when not indicated, and it is shown only along the backbone. In Folk et al. (2024a), clade names from A to H matching nomenclature proposed by Kazempour Osaloo et al. (2005) are reported in parentheses. The sizes of collapsed clades in both trees are not proportional to the number of taxa sampled; see Figure 1 and Folk et al. (2024b) for the extended version of the same trees.

The recovered nuclear phylogeny showed a strong overlap with current phylogenies in terms of clades recovered, especially with that of Folk et al. (2024a; Figure 3), even though there were some major topological differences. However, contrary to Folk et al. (2024a), we obtained higher support, especially within the main clades, and almost all relationships inside the main clades were resolved with high support (LPP = 1). The lower support obtained by Folk et al. (2024a) compared to our study may be attributed not only to the smaller number of targeted loci, but also to the presence of paralogous sequences and genomic conflict, which is difficult to investigate when using a more general or universal bait set, and the smaller capture space (116,680 bp vs. 796,855 bp) of their bait set.

A comparison of the placement of Neo‐*Astragalus* between this and other studies is complicated by differences in taxon sampling. For example, Su et al. (2021) sampled only one species (*A. thurberi*) to represent the more than 450 Neo‐*Astragalus* taxa, which was then resolved as sister to two species (*A. arpilobus* and *A. stalinskyi*) from NW China. Those two species were also sampled by Azani et al. (2017), who included more eastern hemisphere taxa, but they found Neo‐*Astragalus* (10 taxa sampled) to be sister to other species instead. In our study, similar to that of Azani et al. (2017), Neo‐*Astragalus* (five taxa sampled) was placed as sister to Afro‐Mediterranean species and inside the Diholcos clade, indicating that the ancestor of Neo‐*Astragalus* has arisen from annual ancestors of the Diholcos clade. This placement conflicts with the findings of Folk et al. (2024a), which placed Neo‐*Astragalus* as an independent clade along the backbone and sister to a large clade composed of Hypoglottis and Diholcos taxa (Figure 3). The biogeographic results of Folk et al. (2024a) suggested that a single dispersal event from western Asia about 9.8 Mya, thus significantly earlier than previous estimates (Scherson et al., 2008; Azani et al., 2019), was behind the broad ancestral distribution of the Neo‐*Astragalus* clade across the Americas. In our results, North American euploid (*A. americanus*, *A. canadensis*) and circumboreal (*A. laxmannii*) species were nested within eastern hemisphere clades in nuclear (Figure 1; Appendix S2: Figure S2) and chloroplast (Appendix S2: Figure S3) phylogenies consistent with previous studies (Wojciechowski et al., 1999; Azani et al., 2017; Su et al., 2021; Folk et al., 2024a). Our results provide further evidence for the descent of the euploid western hemisphere species from ancestors of the Hamosa and Phaca clades and not being closely related to the Neo‐*Astragalus* clade. Despite being based on a limited sample size (seven out of approximately 300 species), our phylogenetic analysis supports the Astracantha clade, which includes significant components of subalpine and alpine vegetation in the mountainous regions of Southwest Asia (Zohary, 1973). This finding aligns with recent molecular phylogenetic data (Azani et al., 2017, 2019; Folk et al., 2024a), although the clade was either poorly represented in earlier studies (Wojciechowski et al., 1999) or not resolved (Kazempour Osaloo et al., 2003). The clade Pseudosesbanella was recovered in agreement with Su et al. (2021) and Folk et al. (2024a), though it was resolved with very low support in the latter work. The placement of the Glottis clade as sister clade to the rest of Eu‐*Astragalus* was recovered in our study with high support and in agreement with Azani et al. (2017) and Su et al. (2021) (Figure 1). However, the single taxon belonging to the Glottis clade included by Folk et al. (2024a), *A. epiglottis* (syn. *Biserrula epiglottis*; another Glottis member, *A. annularis*, was sampled by the authors but did not meet filtering criteria), was placed outside Eu‐*Astragalus* with very low support (<0.5 LPP, Figure 3). The finding of Folk et al. (2024a) of an unexpected position of *A. epiglottis* outside Eu‐*Astragalus*—more closely related to the Coluteoid clade—echoes the recent segregation of *Biserrula epiglottis* from *Astragalus* (Coulot et al., 2014). However, this placement disagrees with authoritative taxonomic studies (Barneby, 1964; Podlech and Zarre, 2013) and the phylogenetic position of *A. epiglottis* together with *A. annularis* and *A. pelecinus*, as a member of the Glottis clade in the present analyses (Figure 1; Appendix S2: Figures S2 and S3), and with other studies (e.g., Kazempour Osaloo et al., 2003; Wojciechowski, 2005; Azani et al., 2017), where it is nested with strong support (LPP = 1) in the Glottis clade. Members of the Glottis clade with a most likely Mediterranean origin (Azani et al., 2019) are annual plants with very small flowers with a calyx shorter than 4 mm and a standard not exceeding 5 mm (Podlech and Zarre, 2013). The presence of only five fertile stamens in two of three species forming this clade (i.e., *A. pelecinus* and *A. epiglottis*) and a unique type of legume that is falcate but strongly dorsiventrally compressed/flattened in *A. pelecinus* and *A. biserrula*, are other morphological characteristics that are rather unusual within *Astragalus*. However, despite these distinctive traits, the phylogenetic position of this clade within Eu‐*Astragalus* is strongly supported by plastid and nuclear data.

### Cytonuclear discrepancies

We observed some incongruence between nuclear and plastid data (Figure 2A). For example, in the chloroplast tree, the Ophiocarpus clade—represented by four taxa—is monophyletic and sister to the rest of Eu‐*Astragalus*, but according to nuclear data, this clade is polyphyletic and nested in the Hypoglottis clade (Figure 2A). Folk et al. (2024a) included two species belonging to Ophiocarpus, which were placed in two different positions: inside the Astracantha (*A. leucocephalus*, not sampled in our study) and Hypoglottis (*A. hispidulus*, sampled in our study and also placed in Hypoglottis in the nuclear data) clades. Azani et al. (2017) retrieved a monophyletic, well‐supported Ophiocarpus clade (four taxa sampled), but the authors used a combination of nuclear and plastid data (ITS+*matK*). The Ophiocarpus clade is monophyletic in their *matK* gene tree (Azani et al., 2017; Appendix S6) and polyphyletic and nested among Diholcos and Hypoglottis taxa in their ITS tree (Azani et al., 2017; Appendix S5). Our results placed *A. simonii* in the Diholcos clade and sister to Neo‐*Astragalus* in the coalescent‐based and concatenated ML nuclear trees. This placement is incongruent with previous studies and morphology. Still, in our chloroplast phylogeny, the same sample falls in the Trimeniaeus clade, in agreement with the phylogeny of Azani et al. (2017). Here again, in the tree of Azani et al. (2017) based only on the *matK* gene (Azani et al., 2017; Appendix S6), *A. simonii* is located in the Hypoglottis clade (though with low support). Our results also showed that *A. stalinskyi* has an ambiguous position, placed within Hypoglottis (all nuclear‐based phylogenies) or Diholcos clades (chloroplast phylogeny—in agreement with Su et al., 2021). The Diholcos clade, based on nuclear data, was nested inside the Hypoglottis clade (both in coalescent and concatenated ML trees based on orthologous sequences, but see coalescent ASTRAL‐Pro tree based on homologous sequences, where Hypoglottis is monophyletic and sister to Diholcos, inset in Figure 1), while in the chloroplast phylogeny, it was placed as sister to the Astracantha clade (Figure 2A). Additionally, the clades Contortuplicata and Trimeniaeus were placed in different positions in the two trees.

Similar discrepancies are often observed when comparing phylogenies derived from different plant genomic sources and are attributed to reticulate evolution (Soltis and Kuzoff, 1995). In *Astragalus*, except in rare reports based on morphology, hybridization has been regarded as rare or non‐existent due to the breeding biology of the species, assumed to be mostly autogamous (Podlech, 1988; Liston, 1992; Kazemi et al., 2009). However, Watrous and Cane (2011), based on 29 taxa with different distributions, showed that half of the species were self‐compatible, one‐third were obligate outcrossers, and the rest were self‐compatible with outcrossing beneficial. Recent evidence of reticulate evolution and incomplete lineage sorting has been reported (Kazemi et al., 2009; Bartha et al., 2013; Záveská et al., 2019; Maylandt et al., 2024a, 2024b). After finding paralogy in ITS sequences, Bartha et al. (2013) suggested that merging (as opposed to dichotomous splitting of lineages) occurred in sect. *Dissitiflori*. Allopolyploidization played a significant role in the evolution of octoploid populations of *A. onobrychis*, which also resulted in incongruities between nuclear and plastid genomes (Záveská et al., 2019). Cytonuclear discordance has also been observed in *A. sulcatus*, indicating horizontal gene flow from other species in sect. *Dissitiflori* (Maylandt et al., 2024b). *Astragalus dasyanthus* and *A. exscapus* also showed signals of gene flow that occurred in the past (Maylandt et al., 2024a). In our study, although identifying the (extinct or extant) lineages involved in hybridization events may be difficult due to methodological limitations and the selection of appropriate representative taxa for the clades retrieved, the network analysis of the backbone supported the presence of several (at least six) reticulate evolutionary events (Figure 2B; Appendix S2: Figure S6). Almost all those inferred events involved either extinct (ghost) or not sampled lineages and ancestors of current lineages (e.g., between a ghost lineage in Trimeniaeus and the ancestor of Neo‐*Astragalus*, Figure 2B). Interestingly, scenarios that hypothesized different numbers of reticulation events provided some consistent hybridization events (e.g., between Trimeniaeus and Diholcos and Trimeniaeus and a common ancestor of Hypoglottis plus Diholcos clades—including Neo‐*Astragalus*, see Appendix S2: Figure S6), while some other hybridization events were unique for a certain scenario (e.g., between an ancestor of all Meso‐*Astragalus* and Hypoglottis). We believe a larger taxon sampling may provide better estimates and higher consistency of those events. Nevertheless, our analysis supports the hypothesis that past hybridization events and/or incomplete lineage sorting played an important role in the evolution and establishment of several clades inside the genus and is not limited to sect. *Dissitiflori* (Maylandt et al., 2024a, 2024b). The occurrence of those ancient hybridization events, mostly in between Meso‐*Astragalus* clades, together with incomplete lineage sorting, overlaps with the high levels of gene discordance observed along the phylogeny backbone (Figure 1). Therefore, the present study adds increasing evidence of horizontal gene flow in several phylogenetically supported *Astragalus* clades.

## CONCLUSIONS

This study aimed to build a robust phylogeny of the mega‐diverse genus *Astragalus* based on an effective taxon‐specific target enrichment bait set. With our carefully selected sampling, we demonstrated that the method successfully obtained a highly supported backbone of the Astragalean clade with full resolution at the infrageneric relationships in *Astragalus*. Importantly, even with a limited number of sampled taxa, we highlighted conspicuous discrepancies between nuclear and plastid signals in *Astragalus* and provided evidence of reticulate evolution between most of the clades supported by molecular data. As a result, we advocate caution when using combined nuclear‐organellar loci such as ITS+*matK* for phylogenetic inference. This effort represents only a first step toward a fine‐scale resolution of the complex evolutionary history of the lineages in this mega‐genus. Comprehensive taxon sampling to cover all the morphologically and genetically identified clades and subclades in *Astragalus* is necessary to disentangle intrageneric relationships. Based on current molecular and morphological studies, we anticipate that for a comprehensive phylogeny of the genus in the eastern hemisphere (~2600 species), a minimum of about 900 species must be included. Such a sampling effort may represent significant challenges in selecting taxa due to the vast geography, the wide range of chromosome numbers, and the taxonomic representation of *Astragalus* sections. However, these phylogenetic studies will set the basis for studying and understanding the evolution of the largest genus of flowering plants. It will allow us to identify shifts in diversification rate and provide a solid base to study diversification drivers and other important phenomena in a group that impresses for its high diversity.

## AUTHOR CONTRIBUTIONS

D.M.B., D.B., and G.K. conceived the idea. D.B. and D.M.B. performed the experiments and conducted formal analyses. S.Z. and A.L. contributed to species identification and critical revisions. All authors contributed to the discussion and final draft of the manuscript.

## Supporting information


**Table S1.** Taxa originally sampled by Azani et al. ( 2017) and resampled in the present study.
**Table S2.** Additional plastome sequences downloaded from NCBI database used in this study.
**Table S3.** Hybpiper gene recovery statistics.
**Table S4.** Statistics of the orthology inference performed with the monophyletic outgroup (MO) method described by Yang and Smith ( 2014).

**Figure S1**. Number of recovered genes with at least 75% of reference sequence length vs age of herbarium specimen.
**Figure S2**. Concatenated maximum likelihood tree (IQtree) produced by concatenating all sequences in a single supermatrix with a total length of 778,623 bp and 27% missing data.
**Figure S3**. Maximum likelihood (IQtree) species tree inferred from plastome sequences, based on 114,580 aligned columns with 74.6% overall matrix occupancy.
**Figure S4.** PhyParts analysis results mapped on the coalescent‐based species tree obtained with ASTRAL.
**Figure S5.** Quartet sampling probabilities mapped on the coalescent‐based species tree obtained with ASTRAL.
**Figure S6.** PhyloNet analysis to reconstruct different scenarios that involved different numbers of reticulation events ranging from one to six.
**Figure S7.** Total log probability vs number of hybridization events inferred by PhyloNet analysis based on 22 taxa (576 loci).

## Data Availability

Target enrichment data generated for this study can be found in the NCBI BioProject PRJNA1242075 (see Table 1 for SRA accessions). Analyses files are available from the Dryad repository DOI: 10.5061/dryad.79cnp5j7g.
