# Supplementary material for: Building a robust backbone for Astragalus using a clade‐specific target enrichment bait set
Source: Am J Bot. 2025 Aug 19;112(8):e70084. doi: 10.1002/ajb2.70084 (PMC12374571; doi:10.1002/ajb2.70084)
Supplement: Supplementary file 2 — Figure S1. Number of recovered genes with at least 75% of reference sequence length vs age of herbarium specimen. Figure S2. Concatenated maximum likelihood tree (IQtree) produced by concatenating all sequences in a single supermatrix with a total length of 778,623 bp and 27% missing data. Figure S3. Maximum likelihood (IQtree) species tree inferred from plastome sequences, based on 114,580 aligned columns with 74.6% overall matrix occupancy. Figure S4. PhyParts analysis results mapped on the coalescent‐based species tree obtained with ASTRAL. Figure S5. Quartet sampling probabilities mapped on the coalescent‐based species tree obtained with ASTRAL. Figure S6. PhyloNet analysis to reconstruct different scenarios that involved different numbers of reticulation events ranging from one to six. Figure S7. Total log probability vs number of hybridization events inferred by PhyloNet analysis based on 22 taxa (576 loci). [file AJB2-112-e70084-s001.pptx]

## Slide 1
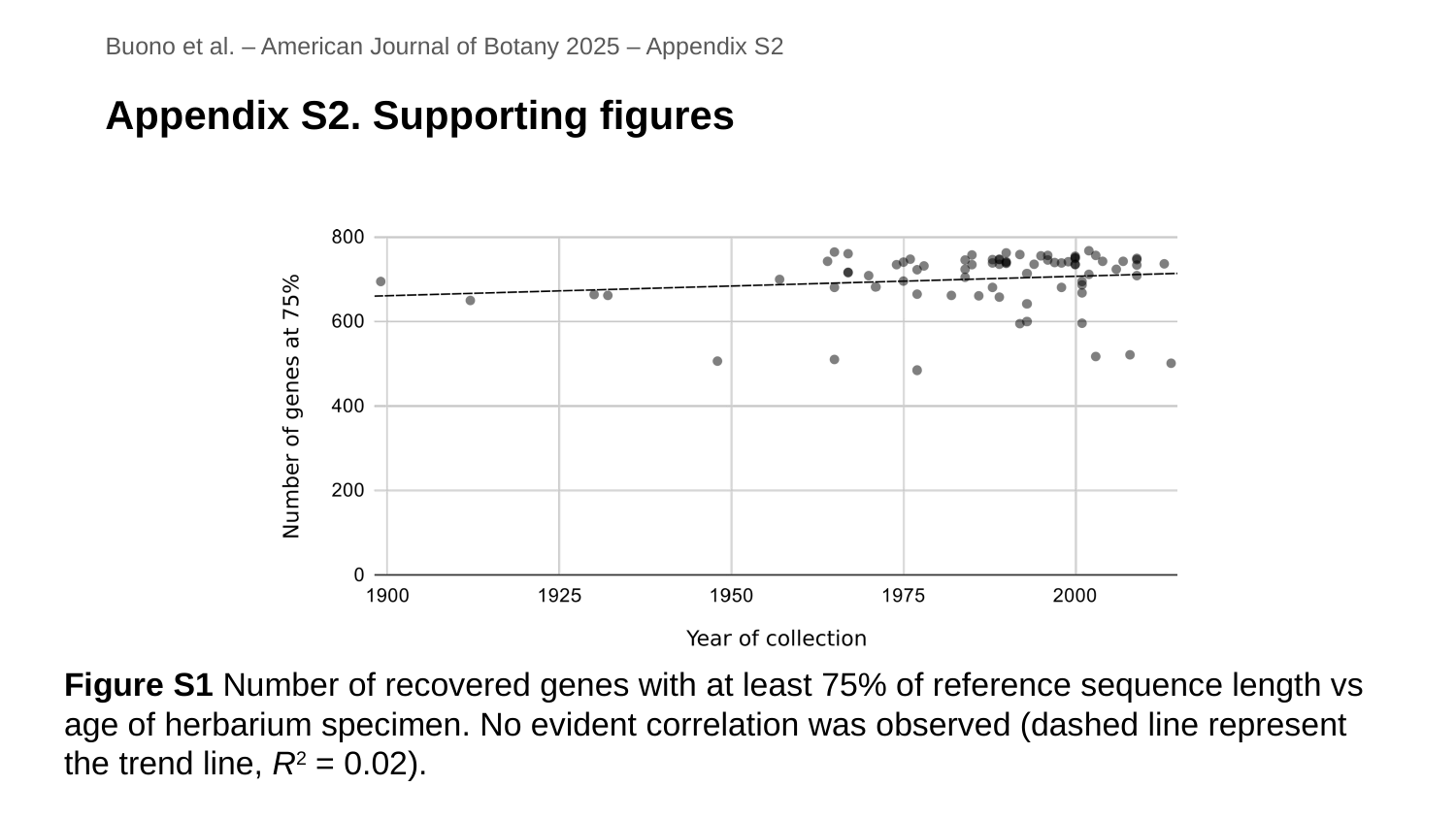

Buono et al. – American Journal of Botany 2025 – Appendix S2
Appendix S2. Supporting figures
# Figure S1 Number of recovered genes with at least 75% of reference sequence length vs age of herbarium specimen. No evident correlation was observed (dashed line represent the trend line, R2 = 0.02).

## Slide 2
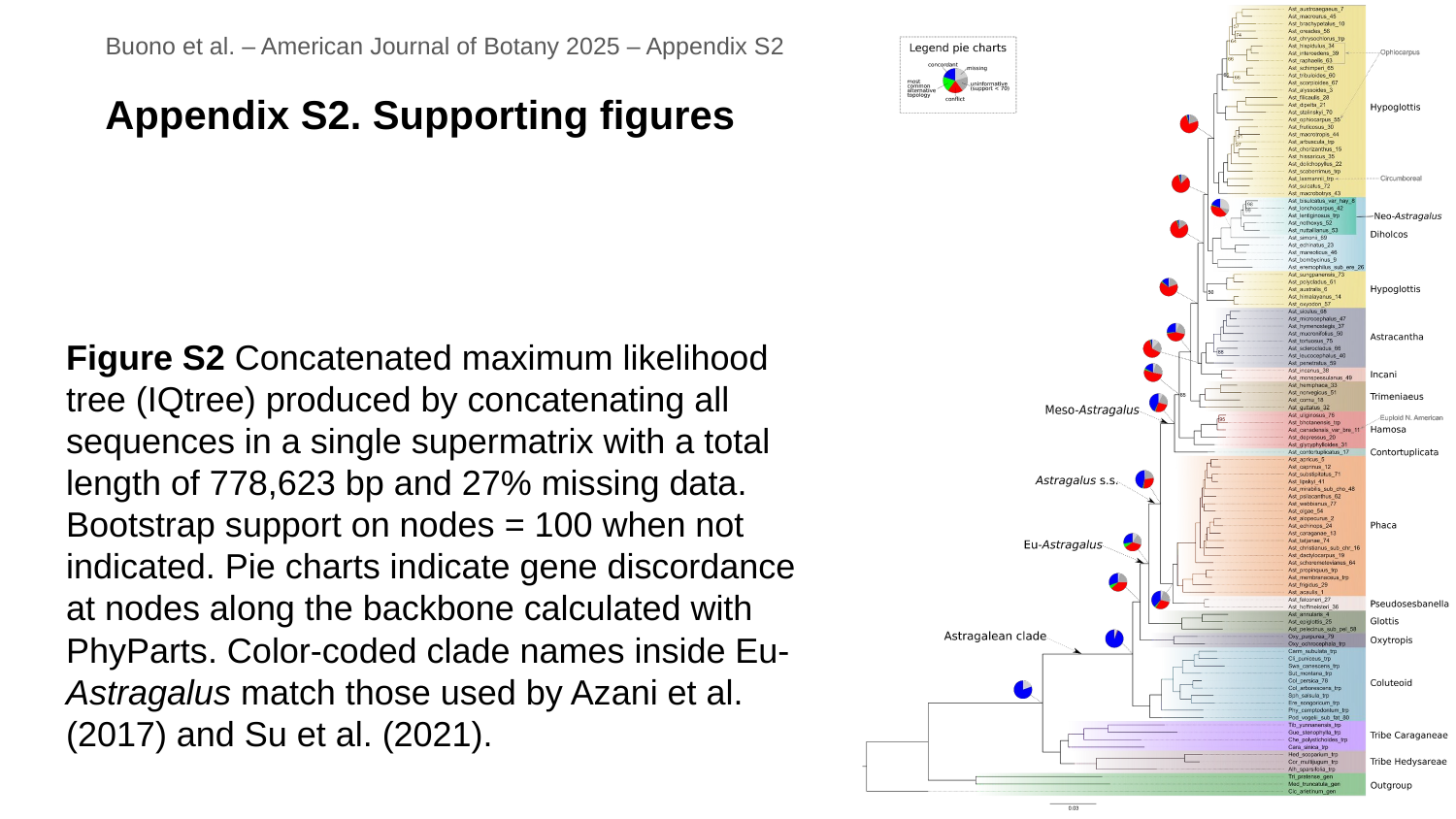

Buono et al. – American Journal of Botany 2025 – Appendix S2
Appendix S2. Supporting figures
# Figure S2 Concatenated maximum likelihood tree (IQtree) produced by concatenating all sequences in a single supermatrix with a total length of 778,623 bp and 27% missing data. Bootstrap support on nodes = 100 when not indicated. Pie charts indicate gene discordance at nodes along the backbone calculated with PhyParts. Color-coded clade names inside Eu-Astragalus match those used by Azani et al. (2017) and Su et al. (2021).

## Slide 3
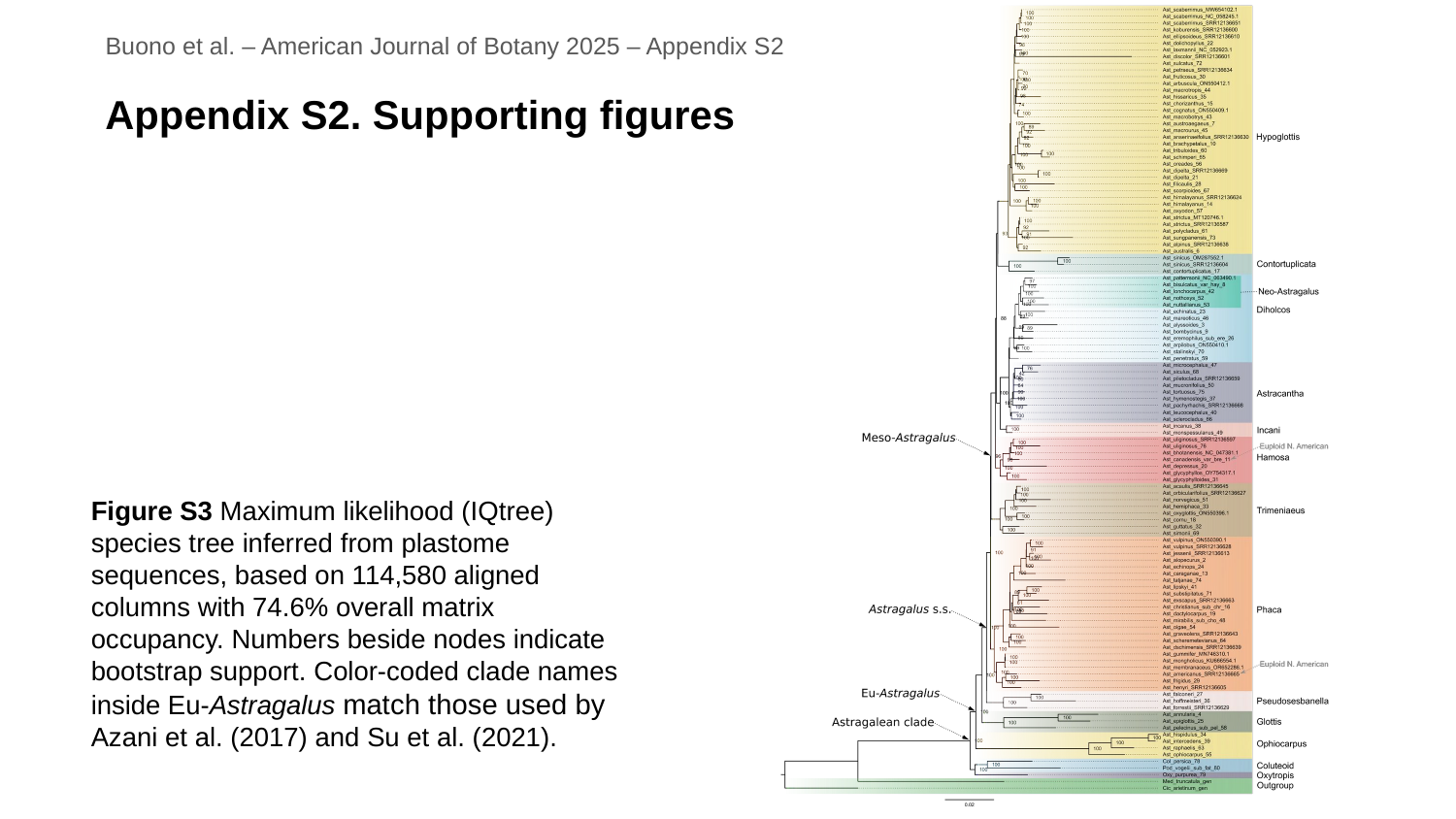

Buono et al. – American Journal of Botany 2025 – Appendix S2
Appendix S2. Supporting figures
# Figure S3 Maximum likelihood (IQtree) species tree inferred from plastome sequences, based on 114,580 aligned columns with 74.6% overall matrix occupancy. Numbers beside nodes indicate bootstrap support. Color-coded clade names inside Eu-Astragalus match those used by Azani et al. (2017) and Su et al. (2021).

## Slide 4
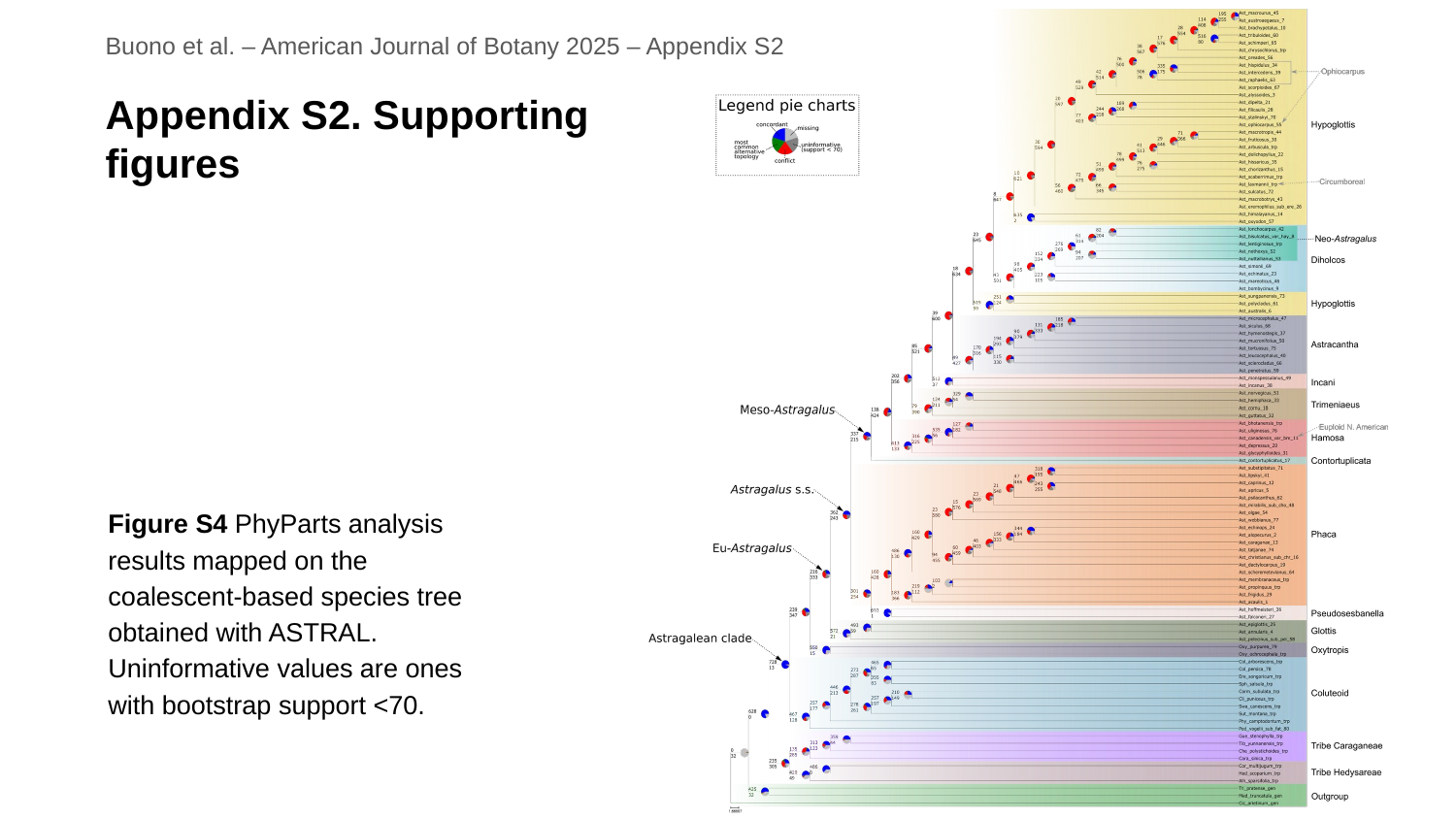

Buono et al. – American Journal of Botany 2025 – Appendix S2
Appendix S2. Supporting figures
# Figure S4 PhyParts analysis results mapped on the coalescent-based species tree obtained with ASTRAL. Uninformative values are ones with bootstrap support <70.

## Slide 5
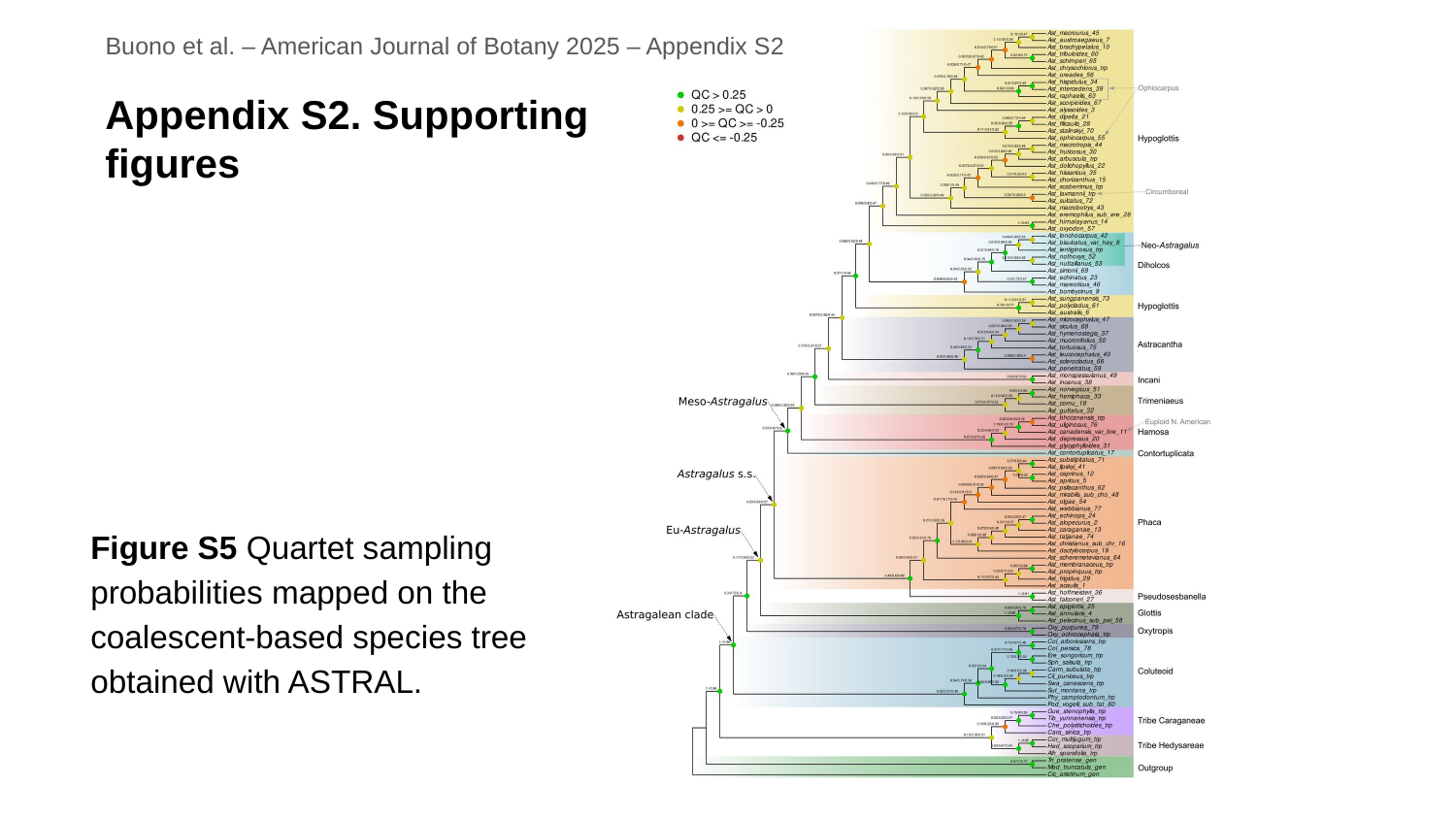

Buono et al. – American Journal of Botany 2025 – Appendix S2
Appendix S2. Supporting figures
# Figure S5 Quartet sampling probabilities mapped on the coalescent-based species tree obtained with ASTRAL.

## Slide 6
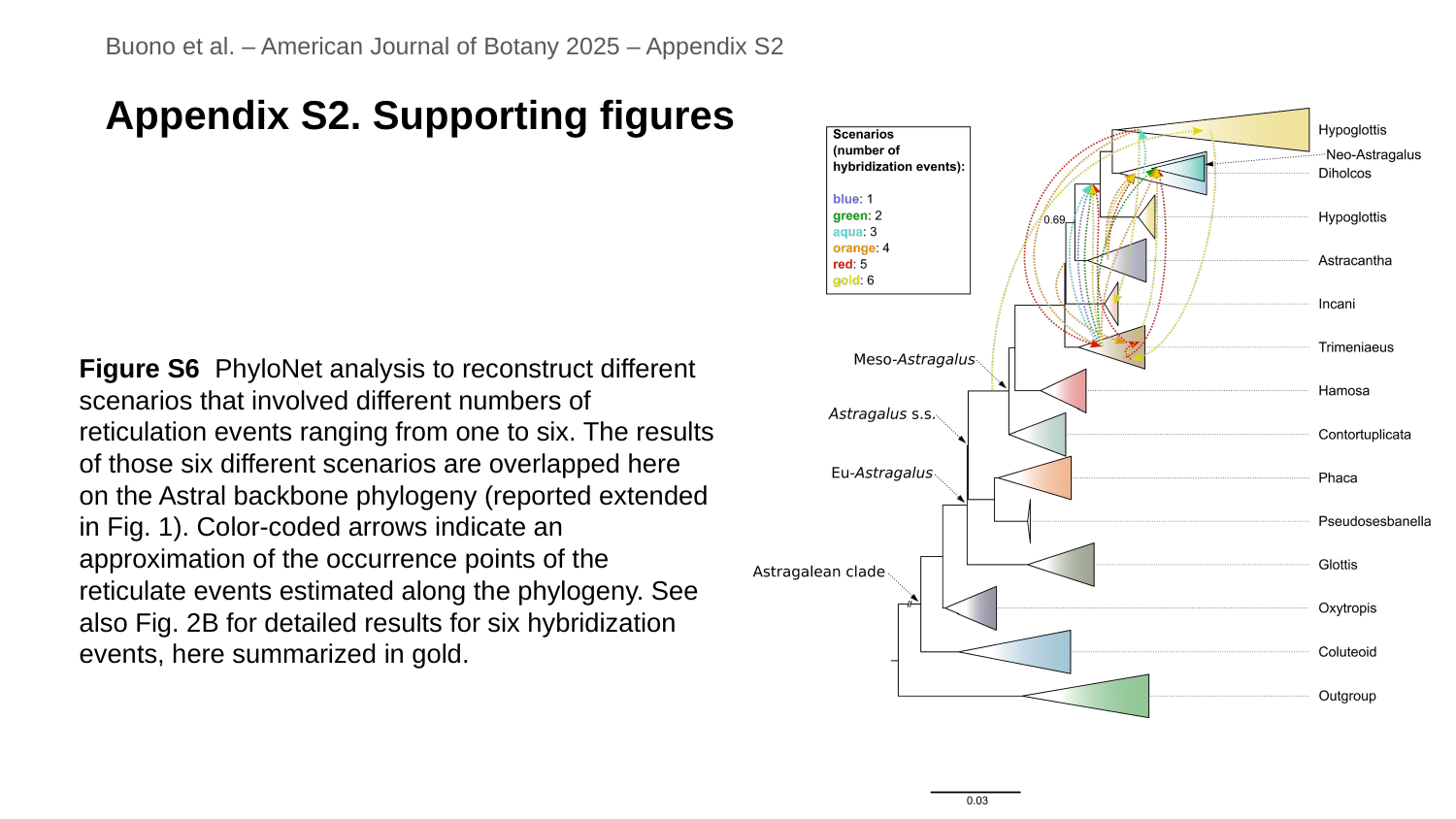

Buono et al. – American Journal of Botany 2025 – Appendix S2
Appendix S2. Supporting figures
# Figure S6 PhyloNet analysis to reconstruct different scenarios that involved different numbers of reticulation events ranging from one to six. The results of those six different scenarios are overlapped here on the Astral backbone phylogeny (reported extended in Fig. 1). Color-coded arrows indicate an approximation of the occurrence points of the reticulate events estimated along the phylogeny. See also Fig. 2B for detailed results for six hybridization events, here summarized in gold.

## Slide 7
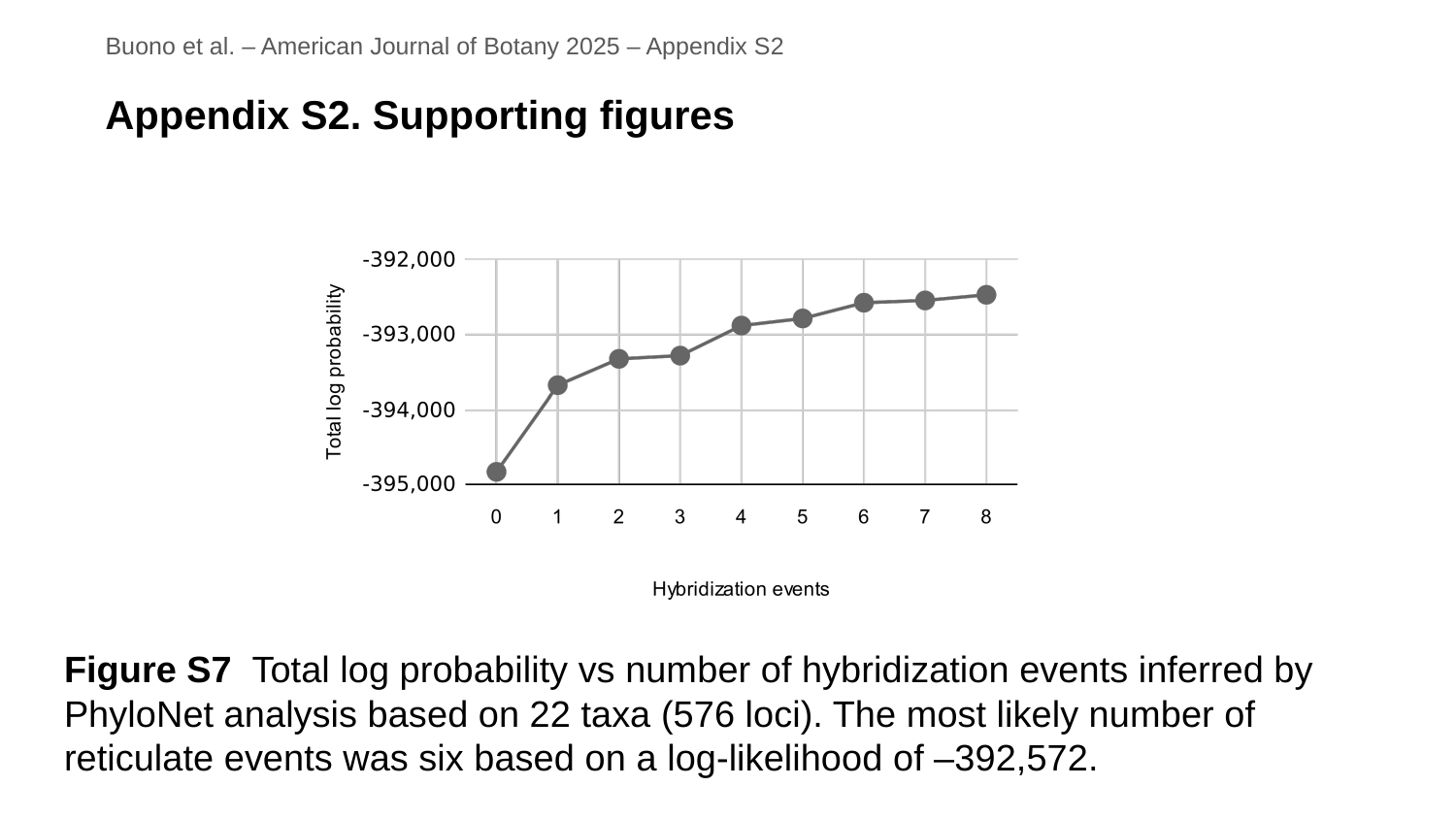

Buono et al. – American Journal of Botany 2025 – Appendix S2
Appendix S2. Supporting figures
# Figure S7 Total log probability vs number of hybridization events inferred by PhyloNet analysis based on 22 taxa (576 loci). The most likely number of reticulate events was six based on a log-likelihood of –392,572.
